# Scratch Morphology Transformation: An Alternative Method of Scratch Processing on Optical Surface

**DOI:** 10.3390/mi12091030

**Published:** 2021-08-27

**Authors:** Guangqi Zhou, Ye Tian, Feng Shi, Ci Song, Guipeng Tie, Shijie Liu, Gang Zhou, Jianda Shao, Zhouling Wu

**Affiliations:** 1 College of Intelligence Science and Technology, National University of Defense Technology, 109 Deya Road, Changsha 410073, China; guangqizhou@foxmail.com (G.Z.); sf.wind@yahoo.com (F.S.); sunicris@163.com (C.S.); tieguipeng@163.com (G.T.); zg2206553079@foxmail.com (G.Z.); 2 Hunan Key Laboratory of Ultra-Precision Machining Technology, Changsha 410073, China; 3 Laboratory of Science and Technology on Integrated Logistics Support, National University of Defense Technology, 109 Deya Road, Changsha 410073, China; 4 Laboratory of Thin Film Optics, Shanghai Institute of Optics and Fine Mechanics, Chinese Academy of Sciences, Jiading, Shanghai 201800, China; shijieliu@siom.ac.cn (S.L.); jdshao@siom.ac.cn (J.S.); 5 Key Laboratory of Nondestructive Testing for Super Smooth Surface of Anhui Province, Hefei 230031, China; zlwu@zc-hightech.com

**Keywords:** optical fabrication, scratch, micro structure fabrication

## Abstract

The scratches on an optical surface can worsen the performance of elements. The normal process method is removing scratches entirely. However, it is a tough and high-cost requirement of removing extremely deep scratches and maintaining all the other excellent indicators at the same time. As the alternative of removing, we propose the method of scratch morphology transformation to diminish the drawbacks induced by scratches. We measure the morphology of scratches, establish the transformation models and transform them to the needed shape. In engineering applications, transformation can solve scratch drawbacks or limitations in an efficient and effective way. Then, residual scratches become acceptable. The transformation can also be amalgamated into the error figuring processes. Typical scratch transforming examples are experimented and AFM measurement is conducted. We explore the rule of scratch morphology transformation by two typical fabrication means: magnetorheological finishing (MRF) and HF etching. This morphology transforming method is an economical alternative for current defect-free fabrication. That will significantly decrease fabrication time, cost and risk, while the optical quality maintain.

## 1. Introduction

In recent years, due to the rapid development of high-power/high-energy laser technology, optics with high laser resistance and precision have been highly needed [1,2]. For most optical substrate materials, scratch is a common defect generated during lapping and polishing [3,4]. To solve the scratch problem, researchers innovated a variety of scratch-less technologies [5,6,7]. With those techniques, it is possible to obtain a scratch-free surface. In engineering fabrication, the normal solution is reducing scratch depth and limiting newborn scratch quantity during the polishing period by environmentally controlling when the surface accuracy and roughness almost fulfill the requirement, a uniform material layer of the total surface, which contains scratches, need to be wiped off entirely before the final process. As a result, scratches are removed when the accuracy indicators reach the standard as well.

However, this method is not a satisfying solution. Firstly, to maintain the high precision in the final fabrication period, the material removal rate must be accurately controlled at a comparatively low level. As a result, the removing efficiency of the scratch layer is low. In our fabrication process of a 430 × 430 mm aspheric lens, the time spent on scratch removing occupies more than 25% of the total polishing period. Besides, edge-cutting optical systems require elements within various indicators [8], and everyone is already challenging for the current fabrication level. Removing material for a long time also enlarges the edge effect [9], generates more mid-spatial error [10] and may ruin the surface roughness. In short, the requirement of being defect-free also influences the ceiling fabrication level of other indicators. Last but not least, actual situations are often twisted. Sometimes, there may exist a single extremely deep scratch on the surface, due to failures in former process. It will cost plenty of time to obtain a defect-free surface before starting the final polishing process [11]. In other awkward occasions, an accidental newborn scratch can ruin the entire fine polishing period. Frankly speaking, in engineering fabrication, pursuing a defect-free surface is risky and costs loads of money as well as time, so scratch morphology transformation is proposed in this paper.

Some researchers have tried to process residual scratches [12], aiming at increasing laser-induced damage threshold (LIDT). Additionally, they found scratch morphology greatly influences LIDT [13,14]. Once the scratches are passivated, their influence on LIDT will be dramatically reduced to the same level of normal surfaces. Then, the residual scratches are acceptable to be reserved on the optical surface. This residual scratch process solution can go further within the deterministic process method.

Like deterministic surface error figuring [15], scratch morphology can also be quantified and regarded as a fabrication indicator. We can measure the morphology of a scratch and abstract the geometry characteristic. We can establish its removal model and deterministically transform the scratch to the shape as we want. Furthermore, researchers can combine the controllable and deterministic transform process with the surface error figuring or roughness improving technology in the entire process design. That allows them to innovate and optimize the whole process more efficiently and economically.

**Figure 1:**
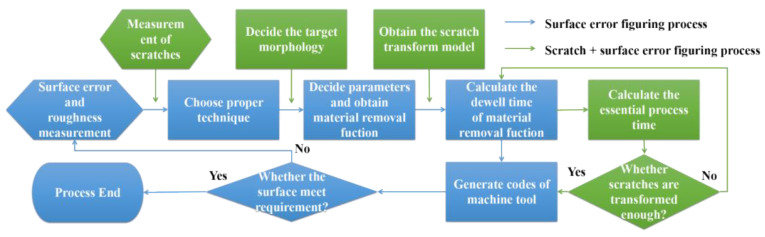
The combined process of scratch transform and surface error figuring.

The conventional process of deterministic surface error figuring [16] is drawn in Figure 1 as the blue path. The scratch-related green path is added, and we combined two fabrication aims into one process method.

In this paper, we propose as well as discuss the scratch morphology transformation method and exhibit two transforming examples using MRF and HF etching. To prove the effect of the new method, a light field distribution simulation and Scanning Electron Microscope-Energy Dispersive Spectrometer (SEM-EDS) are conducted. Their results show that the drawbacks of residual scratches are reduced by transformation. In conclusion, the transforming method is a possible solution to treat scratches at a high efficiency and low cost.

## 2. Scratch Morphology Transformation Examples of MRF and HF Etching

### 2.1. Morphyolog Evolution of MRF Transform

Two fused silica samples are super-polished and few coarse particles are mixed into polishing powder to generate scratches naturally [17]. Especially, we select and locate two scratches which are not very deep. Because once the depth is larger than 1 μm, small removal amounts cannot reform them obviously. We tried to control MRF and HF etching processes in small removal amounts, and observed frequently to search slight changes. The scratch profile measurement was conducted utilizing an atomic force microscope (Bruker^®^, Dimension icon, Tapping Mode, scanned area 30 μm × 30 μm).

**Figure 2:**
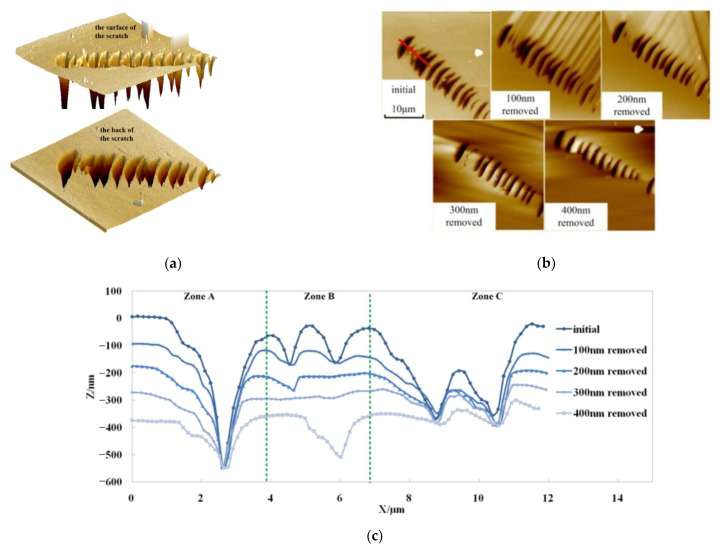
Defect morphology evolution during MRF transformation process. (**a**) 3D AFM picture; (**b**) 2D AFM picture; (**c**) section profile curves of the red line in (**a**).

We conduct MRF experiments on Sample 1. MRF is a process in which magnetorheological fluid circulates into the polishing area. It becomes a viscoplastic Bingham medium under the action of high intensity gradient magnetic fields, forming a “flexible polishing mold” that can adapt to the workpiece shape and remove the workpiece material by plastic shear. HF acid etching is a process of material removal by reacting HF acid with a fused silicon material. To observe the transformation process, the sample was polished layer by layer using MRF. All the AFM results are shown in Figure 2.

Three typical evolution phenomena are observed, and they are illustrated in Zone A, B and C, shown in Figure 2b, and discussed below.

Zone A shows a V-bottom pit, representing the shape of most Hertz scratch pits and constant scratches [17,18]. Zone B shows that the defects were completely removed after polishing at 300 nm, which verified the defect removal capability of MRF. However, after the final polishing, new defects were exposed, indicating that the defect pits of the same scratch are not necessarily buried in the same depth. In this case, if a defect-free surface is required, a large amount of time will be spent polishing at extremely deep defects. Zone C indicates that MRF has good defect removal ability. The profile data is recorded in Table 1.

### 2.2. Analysis and Modeling of MRF

**Figure 3:**
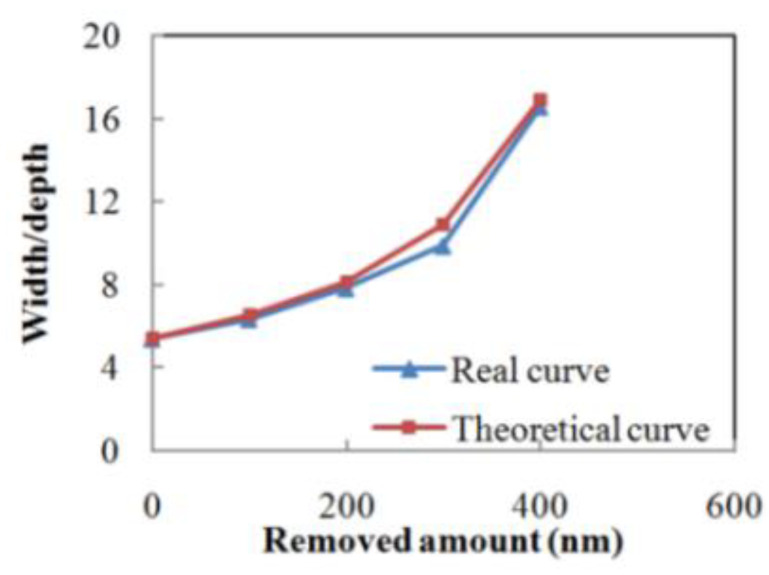
Width/depth ratio transformation of V-bottom pit during MRF.

Based on the profile data, we can figure out that during the MRF process, the width of V-bottom pit hardly changes while the depth decreases with the process going on (the decrease amount numerically equals material removed amount). In short, the defect profile is blunted by the MRF polishing. The width/depth ratio (W/D, R) of the pit could be described as (1):

(1) $$R=\frac{W}{D}=\frac{W_{0}}{D_{0}-\Delta D},\Delta D=\varepsilon t$$

where $W_{0}$ is initial width, $D_{0}$ is initial depth, ε is material removal rate and t is process time. Figure 3 illustrates both the theoretical and measured width/depth ratio evolution curve. The theoretical curve is based on Equation (1).

In Zone B, the initial pits disappear entirely when 300 nm material is removed. However, a defect pit was newly exposed after the last process. This pit is the residue of the last pad polishing process and buried in subsurface. It proves that even a single scratch contains defect pits in various depth layers. Therefore, when we are pursuing a defect-free surface, it will take a large proportion of process time to remove a few extremely deep pits.

Zone C is a pair of two nearby pits. The pits have a similar shape to Zone A, except they have a small partition between each other. This partition is not polished until its top reaches the horizontal level of the surface.

The test results show that MRF only removes ceiling material and keeps the width of sunken parts, such as Hertz scratch pits and constant scratches. This is due to the hydrodynamic pressure changes near the pit edges and a stronger shear force. The material removal rate on the edge is higher than on the plane [19].

### 2.3. Morphology Evolution of HF Etching Transform

To obtain defect profile evolution during HF etching, a series of etching processes are conducted. The HF concentration was 5 wt.%, corresponding to the material removal rate which was 20 to 40 nm per minute. Every etching took 15 min.

**Figure 4:**
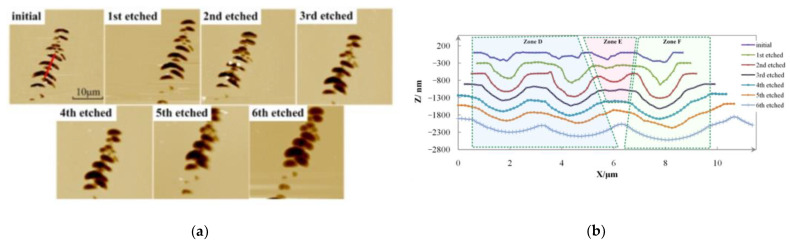
Defect’s morphology evolution during HF etching transformation process. (**a**) AFM pictures; (**b**) section profile curves.

The real profile evolution and transformation efficiency model can be established based on the results. The profile evolution has been measured by AFM and shown in Figure 4a. Profile curves of a pit array (red line in Figure 4a) is drawn in Figure 4b and been divided into Zone D, E and F, in three parts. Zone D and F have a similar transformation route, and the measured data of Zone D is showed in Table 2.

Zone D and F transform similarly, so we only discuss the profile evolution of the left pit in Zone, D. Table 2 lists the profile evolution.

### 2.4. Analysis and Modeling of HF Etching

It could be figured out from the measurement results that:

(1) at the beginning, the initial depth is unreliable, since the defect pits are too narrow to be detected by the AFM probe;
(2) after the first etching, pit valleys are partly opened, so the detected depth increases. Besides, those sharp edges and angles become blunt and rounded. The second etching opens the defects further and the depths keep the same in following measurements;
(3) the fifth and sixth measurements show the depth shrinks since the boarders of nearby pit cross and the partition disappears. This phenomenon exists only for pit arrays;
(4) in contrast to depth, the width increases constantly, so the W/D keeps increasing.

Because the etching increases the defect width and keeps the depth, the defects have larger W/D and become passivated.

**Figure 5:**
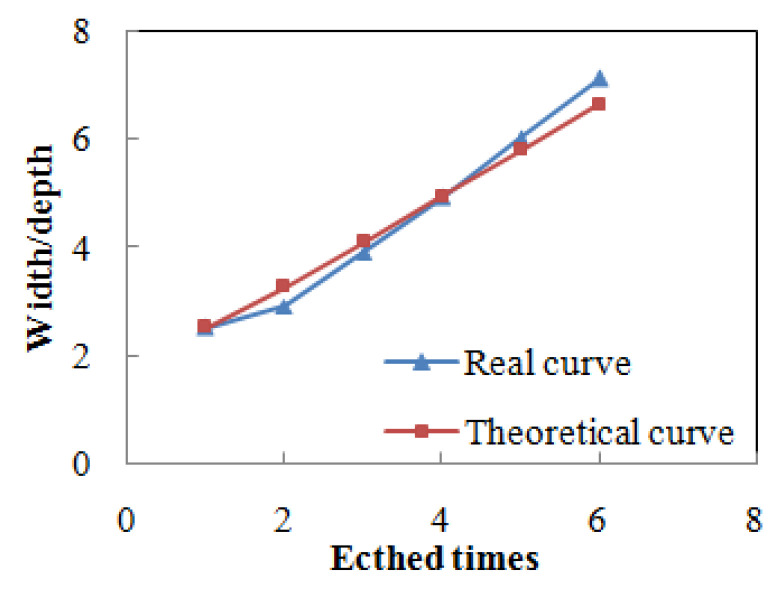
Transformation process of HF etching.

Like the results in reference [20], the bottom and the sides of pits at almost the same rate, and the horizontal expanding rate, is theoretically one times higher than the orthogonal direction. Defect depth remains the same before the borders of nearby pits intersect together. The width/depth evolution model is given as Equation (2)

(2) $$R=\frac{W}{D}=\frac{W_{1}+2\varepsilon_{HF}t}{D_{1}}$$

where W_1_ and D_1_ are the width and depth after MRF polishing, respectively. *ε_HF_* is the material removal rate of HF etching and t is the etching process time. In the initial state, the defect depth has not been expanded and the depth measurement results are inaccurate, so the data in the initial state are not considered. It is clear that the transformation extent is in direct proportion to the process time. The gradient of the function line is two times the removal rate, and the intersection of the function line with the Y axis is the W/D of the defect after polishing, as shown in Figure 5. The theoretical curve is based on Equation (2)

Zone E—the depth of Zone E is not exactly detected until the third etching. In the fourth etching, the pit directly disappeared. Because the pit of Zone E is becoming wider and the two pits beside it which are also expanding, the two heaves of the third line are etched off after the fourth process.

## 3. The Effect of Transformation

### 3.1. Light Field Enhancement Decreasing after Transform

**Figure 6:**
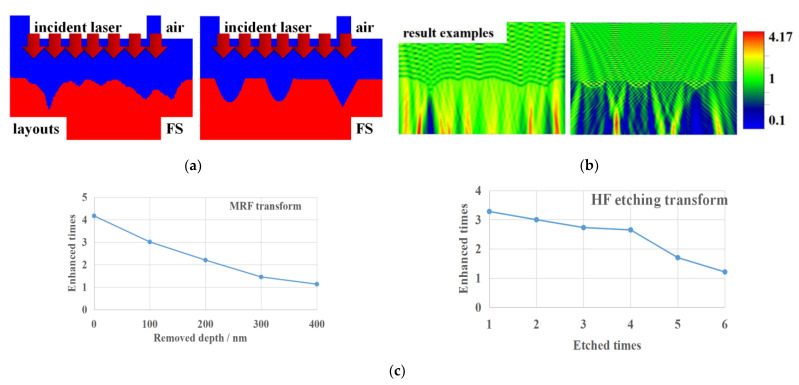
FDTD simulation of scratches; (**a**) model layouts; (**b**) simulation results; (**c**) light field enhancement evolution trends.

Scratches significantly influence the nearby light field distribution and generate intensity enhancement. The enhancing extent is directly related to the scratch morphology [14,21]. Utilizing finite difference time domain (FDTD) simulation, we established a light field distribution model of the scratches in Section 2. The simulation area was set as 5 μm × 5 μm × 3 μm, and there were 0.5 μm thick air interlayers on the front and rear surfaces of the workpiece. The 355 nm wavelength laser was on the left side of the simulation area. Five scratch calculation models were constructed. The scratch was 1 μm to 3 μm in width, and each scratch gradually changed from shallow to deep. There were twenty-six kinds of width to depth ratios which were taken to increase from 1 to 11. The model layouts are shown in Figure 6a, and the simulation parameters are listed in Table 3. We obtain the ratio between the maximum value of different treated light fields in the same region and the standard light intensity. The results are shown in Figure 6c.

Results are shown in Figure 6b, and red areas represent the enhanced points. After a series of simulations, the trends of enhanced rate are drawn in Figure 6c. With the scratches transformed, the enhanced rates decline for both processes. The results of FDTD simulation illustrate that the scratch transform is effective to reduce the light enhancement. The closer to one of the light enhancement, the closer to the defect-free surface of the laser damage ability of the optical element surface. Although scratches still exist on the surface, the light field enhancement almost reaches one, which is the same level as the normal surface. For deep scratches, transforming can save large amount of processing time compared with removing. 

### 3.2. Chemical Impurity Cleaning after HF Transform

**Figure 7:**
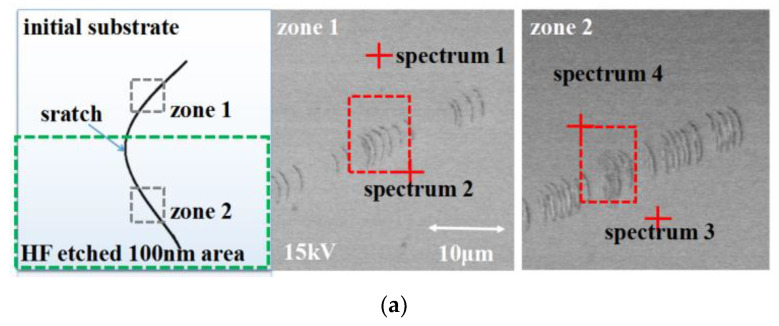

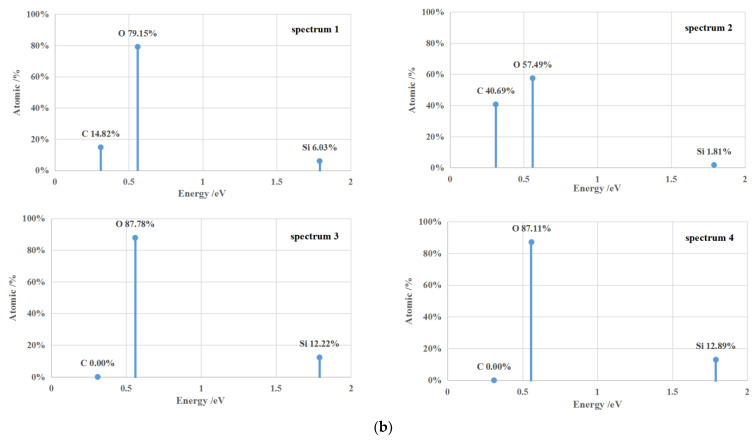
The chemical element changing after HF etching, tested by EDS. (**a**) EDS test area; (**b**) element content.

Impurities are buried in scratches and HF transform can uncover the scratch. We detect chemical element evolution after the HF transforming with the EDS test. On the test sample, a long scratch crosses the entire surface. The surface is ultrasonically cleaned by deionized water and divided into two halves. Then, the bottom half is HF etched for 100 nm material. After that, we observed the scratch in two zones and detected chemical elements. The results are shown in Figure 7a.

On the initial surface, the carbon is detected as 14.82 atomic% on the normal area and 40.69% on the scratch area, respectively. After a HF etching transform, all the carbon impurities are removed in both areas. Besides, the chemical compositions of the scratch area and normal surface become the same.

Before transforming, although the surface had been ultrasonically cleaned, the scratch area contained two times more impurities than normal surfaces. After the transformation, although the scratches still exist, the impurities in scratches are entirely removed just as in the normal surface zone. Therefore, impurities can be thoroughly cleaned after transforming.

## 4. Conclusions

In this paper, we propose scratch transformation as an alternative method to treat scratches on optical surfaces. Scratches are accurately measured and can be transformed to the shape we need. Scratch-less technologies can be utilized in scratch transformation. As examples, we applied MRF and HF etching to transform scratches. The scratch morphology transforming evolution processes are observed and models are established. The scratch can also be transformed to meet the application needs. In this way, scratches can be treated as a normal indicator just like surface error and roughness. Additionally, it can be improved with a deterministic method. Then, FDTD simulations and EDS analysis are conducted to prove that the drawbacks of scratches can be restrained by transformation. As a conclusion, residual scratches are acceptable with proper transformation.

## Figures and Tables

**Table 1:** Profile evolution of a V-bottom pit during MRF.

Removed Amount	Width (μm)	Depth (nm)	Width/Depth
0	2.955	548.61	5.39
100 nm	2.865	451.65	6.34
200 nm	2.913	371.91	7.83
300 nm	2.733	276.61	9.88
400 nm	2.814	169.71	16.58

**Table 2:** Profile data during HF etching of the left pit in Zone, D.

State	Width/nm	Depth/nm	W/D
initial	1380	272	5.1
1st etched	1500	611	2.5
2nd etched	2040	711	2.9
3rd etched	2750	713	3.9
4th etched	3250	713	4.9
5th etched	4160	693	6.0
6th etched	4760	670	7.1

**Table 3:** FDTD simulation parameters.

Parameters	Value
Simulation area	12 μm × 6 μm
Discrete space interval	10 nm
Discrete time interval	5 × 10^−17^ s
Time length	10^−13^ s
Boundary condition	PML
Steps	2000

## Data Availability

The data presented in this study are available on request from the corresponding author. The data are not publicly available due to the data also forms part of an ongoing study.
